# Inhibition of Hepatocyte Apoptosis: An Important Mechanism of Corn Peptides Attenuating Liver Injury Induced by Ethanol

**DOI:** 10.3390/ijms160922062

**Published:** 2015-09-11

**Authors:** Zhili Ma, Tao Hou, Wen Shi, Weiwei Liu, Hui He

**Affiliations:** College of Food Science and Technology, Huazhong Agricultural University & Key Laboratory of Environment Correlative Dietology, Ministry of Education, Wuhan 430070, China; E-Mails: mazhili@webmail.hzau.edu.cn (Z.M.); mrhoutao@webmail.hzau.edu.cn (T.H.); trshiwenqqq@163.com (W.S.); liuweiweifly@sina.com (W.L.)

**Keywords:** corn peptides (CPs), pentapeptide, ethanol, apoptosis

## Abstract

In this study, the effects of mixed corn peptides and synthetic pentapeptide (QLLPF) on hepatocyte apoptosis induced by ethanol were investigated *in vivo*. QLLPF, was previously characterized from corn protein hydrolysis, which had been shown to exert good facilitating alcohol metabolism activity. Mice were pre-treated with the mixed corn peptides and the pentapeptide for 1 week and then treated with ethanol. After treatment of three weeks, the biochemical indices and the key ethanol metabolizing enzymes, the serum TNF-α, liver TGF-β1 concentrations and the protein expressions related to apoptosis were determined. We found that the Bcl-2, Bax and cytochrome c expressions in the intrinsic pathway and the Fas, FasL and NF-κB expressions in the extrinsic pathway together with higher TNF-α and TGF-β1 concentrations were reversed compared with the model group by both the mixed corn peptides and the pentapeptide. The activation of caspase3 was also suppressed. Additionally, apoptosis was further confirmed with terminal deoxynucleotidyl transferase dUTP nick end labeling (TUNEL) and the TUNEL assay demonstrated peptides suppressed hepatocyte apoptosis. Our results suggest that apoptosis induced by ethanol is alleviated in response to the treatment of corn peptides, potentially due to reversing the related protein expression.

## 1. Introduction

Chronic excessive ethanol intake has been known as a main cause of alcoholic liver disease which is one of the most serious liver disorders [1]. Alcohol consumption can lead to cell apoptosis in liver [2], which has been also observed in an alcoholic liver injury experiment [3] and in a clinical experiment [4]. There are two main signaling pathways of apoptosis: extrinsic pathway which is mediated by death receptors and Bcl-2-controlled intrinsic pathway [5]. Also, the intrinsic pathway is often called mitochondria-mediated death pathway [6,7,8]. Acute ethanol induces membrane permeability transition (MPT) via oxidative stress, and the MPT mediates mitochondrial pathway of apoptosis in hepatocytes exposed to acute ethanol [9]. Moreover, results of another study support that ethanol induces apoptosis via two different pathways: MPT and up-regulation of the expression of CD95-Fas ligand [10], suggesting that apoptosis induced by ethanol is related with both intrinsic and extrinsic pathways.

In recent years, the corn protein is acknowledged to be a good source of bioactive peptides with a broad spectrum of biological activities, such as inhibiting angiotensin I converting enzyme (ACE-I) [11], free radical scavenging activities [12,13], facilitating alcohol metabolism [14], and bile acid binding capacity [15]. In our previous studies, corn peptides (CPs) have been found to provide significant protection against liver injuries in mice induced by alcohol, Bacillus Calmette-Guerin/lipopolysaccharide, carbon tetrachloride and thioacetamide-induced liver fibrosis [16,17,18,19]. Another study also found that corn oligopeptides have a significant protective effect on early alcoholic liver injury in rats [20]. Wu *et al.* have reported the protective effects of corn peptides against alcoholic liver injury in men with chronic alcohol consumption at the dose of 4 g/day and indicated that CPs may have protective effects on alcohol-induced liver damage via modulation of lipid metabolism and oxidative stress [21]. The results from these studies demonstrate that CPs can prevent ethanol-triggered malonaldehyde (MDA) generation and restore the antioxidant capability of hepatocytes through increasing glutathione (GSH) content which has been reduced in the injuries. It is known that oxidative stress plays an important role in the development of alcoholic liver diseases (ALD) [22]. Decreases in intracellular GSH have been shown to be an early event in apoptosis [23]. Production of reactive oxygen species may lead to mitochondrial damage that produces a leak of cytochrome c (cyt c), which activates caspases and causes apoptosis [24]. There is some research about anti-apoptotic effect of bioactive compounds. It has been reported that l-theanine inhibited ethanol-induced L02 cell apoptosis by the experiments of DAPI staining, pro-caspase3 level and PARP cleavage determination [25]. Recent studies have demonstrated that Aplysin could improve the histopathological damages and serum biochemical indices, influence ethanol metabolizing enzymes and inhibit the liver apoptosis, which ultimately prevented and protected the ethanol-induced liver injury [26]. However, in the previous study, the research related to the hepatoprotective effect of corn peptides mainly concentrated on the evaluation of biochemical indices and histopathological analysis. The mechanism associated with the protection of apoptosis by corn peptides has not yet been addressed. In this study, besides the detection of the key metabolic enzymes and oxidative damage, we will focus on whether the corn peptides could inhibit hepatocyte apoptosis induced by ethanol, to explore the further mechanisms of corn peptides in exerting a hepatoprotective effect. In addition, the hepatoprotective effect of a corn-derived peptide QLLPF identified from the mixed corn peptides [27] was also investigated.

## 2. Results

### 2.1. Effects of the Peptides on the Body Weights and Liver Index

**Figure 1:**
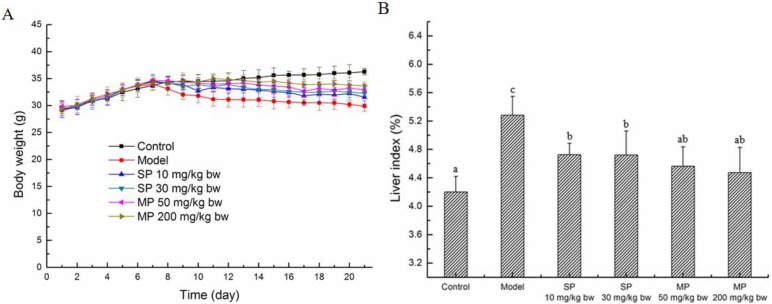
Body weight (**A**) and the liver index (%) (**B**) of mice in each group. Values not sharing a common letter (a, b, c) differ significantly at *p* < 0.05. SP, synthetic pentapeptide; MP, mixed corn peptides.

The body weights of the mice were measured every day during the experiment period. As shown in Figure 1A, the average body weights of all the groups increased during the first seven days, whereas that of the model group and the peptide-treated groups decreased after treatment with ethanol. Compared with the ethanol treated group, the body weights of the groups treated with the synthetic pentapeptide QLLPF and the mixed corn peptides increased during the treatment with ethanol. In addition, Figure 1B showed that ethanol intake led to the significant increase in the liver index (*p* < 0.05), and the pentapeptide QLLPF and the mixed corn peptides suppressed these adverse effects. Specifically, the liver index of the two mixed corn peptides groups have been reduced to the normal level.

### 2.2. Effects of the Peptides on the Activities of Serum Alanine Aminotransferase (ALT) and Aspartate Aminotransferase (AST), the Levels of Hepatic Malonaldehyde (MDA) and Glutathione (GSH)

The serum ALT and AST activities are the biomarkers in liver injury. As shown in Table 1, the model group showed a significant increase over the control group in the activities of ALT and AST (*p* < 0.05). The two mixed peptides groups showed a significantly lower level of AST than the model group (*p* < 0.05) and the level returned to normal at both 50 and 200 mg/kg doses. The levels of MDA and GSH in each group are also listed in Table 1, and the ethanol intake led to a marked increase of MDA and a marked decrease of GSH in the model group (*p* < 0.05). The reduction of MDA and the enhancement of GSH (*p* < 0.05) were indications for the alleviation of liver injury in all the peptides-treated groups. As to the levels of MDA and GSH, the two pentapeptides groups showed a significant improvement over the corresponding model groups. Generally, the mixed peptides groups exerted a better effect than the pentapeptides groups.

**Table 1:** Effects of peptides on serum AST, ALT activities, hepatic MDA and GSH.

Groups	ALT (IU/l)	AST (IU/l)	MDA (nmol/mg pro)	GSH (μmol/g pro)
Control	7.77 ± 3.52 ^a^	10.66 ± 2.03 ^a^	2.51 ± 0.25 ^a^	2.85 ± 0.35 ^b^
Model	12.75 ± 4.40 ^b^	17.21 ± 8.97 ^b^	5.08 ± 0.45 ^d^	2.27 ± 0.16 ^a^
SP 10 mg/kg bw	10.45 ± 3.52 ^ab^	15.21 ± 2.77 ^ab^	3.47 ± 0.34 ^c^	3.09 ± 0.45 ^b^^c^
SP 30 mg/kg bw	9.56 ± 2.22 ^ab^	12.42 ± 3.52 ^ab^	3.40 ± 0.32 ^c^	3.50 ± 0.39 ^c^
MP 50 mg/kg bw	8.93 ± 2.34 ^ab^	11.68 ± 2.05 ^a^	3.15 ± 0.40 ^b^^c^	3.31 ± 0.33 ^b^^c^
MP 200 mg/kg bw	8.53 ± 2.03 ^ab^	11.53 ± 1.54 ^a^	2.84 ± 0.44 ^ab^	3.54 ± 0.27 ^c^

^a,b,c,d^ Values not sharing a common superscript letter differ significantly at *p* < 0.05. SP, synthetic pentapeptide; MP, mixed corn peptides.

### 2.3. Effects of the Peptides on Key Ethanol Metabolizing Enzymes

**Figure 2:**
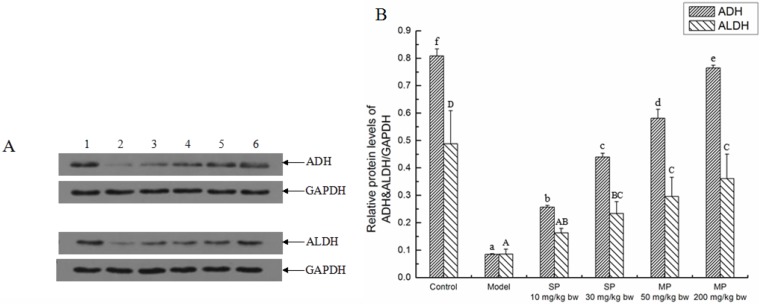
The relative protein expressions of ADH and ALDH in each group. (**A**) Representative western blots for ADH and ALDH, GAPDH was used as internal control for liver tissue; (**B**) Relative protein levels of ADH & ALDH/GAPDH. Values not sharing a common letter (a, b, c, d, e, f) differ significantly of relative protein level of ADH/GAPDH at *p* < 0.05. Values not sharing a common letter (A, B, C, D) differ significantly of relative protein level of ALDH/GAPDH at *p* < 0.05. SP, synthetic pentapeptide; MP, mixed corn peptides. Numbers 1–6 indicate groups of Control, Model, SP-10 mg/kg bw, SP-30 mg/kg bw, MP-50 mg/kg bw and MP-200 mg/kg bw, separately.

Alcohol dehydrogenase (ADH) and aldehyde dehydrogenase (ALDH) are the key enzymes of ADH system of ethanol metabolism. As illustrated in Figure 2, the expression levels of ADH and ALDH were significantly down-regulated when compared with their corresponding control groups, whereas the levels of those were significantly up-regulated in response to the synthetic pentapeptide QLLPF and the mixed corn peptides except the level of ALDH in the SP-10 mg/kg bw group.

### 2.4. Effects of the Peptides on Cytokines TNF-α, TGF-β1

**Figure 3:**
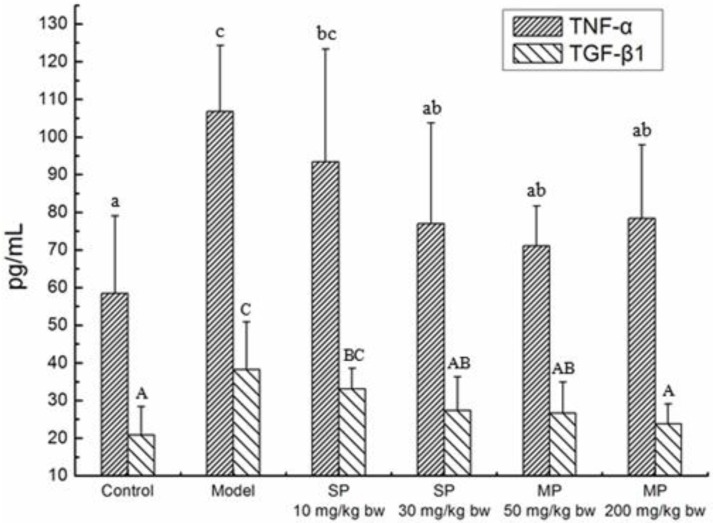
The concentrations of serum TNF-α and the liver TGF-β1 in each group. Values not sharing a common letter (a, b, c) differ significantly of TNF-α level at *p* < 0.05. Values not sharing a common letter (A, B, C) differ significantly of TGF-β1 level at *p* < 0.05. SP, synthetic pentapeptide; MP, mixed corn peptides.

The concentrations of TNF-α and TGF-β1 in each group are shown in Figure 3. In the control group, the concentrations of TNF-α and TGF-β1 were 58.52 and 20.86 pg/mL, respectively. The concentrations were significantly increased to 106.89 and 38.24 pg/mL in the model groups as compared to their respective control levels (*p* < 0.05). However, the levels of the two cytokines showed a tendency to decrease during the treatment of the synthetic pentapeptide QLLPF and the mixed corn peptides. Additionally, the levels of TNF-α and TGF-β1 in all the peptide-treated groups except the low-dose synthetic pentapeptide group of 10 mg/kg bw significantly decreased as compared to the model levels (*p* < 0.05) and returned to the normal level.

### 2.5. Effects of the Peptides on Apoptotic Pathway in Ethanol-Treated Mice

#### 2.5.1. Effects of the Peptides on MnSOD

**Figure 4:**
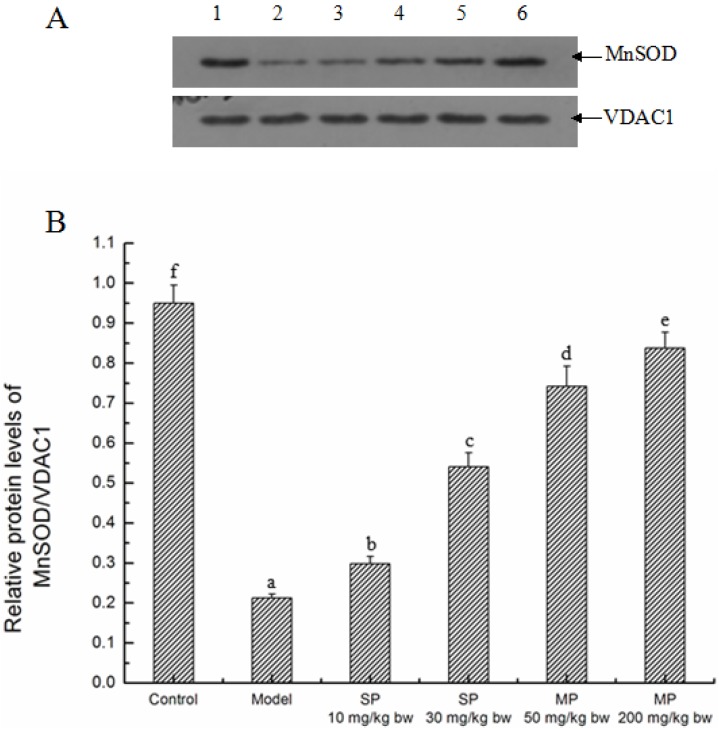
The relative protein expressions of MnSOD in each group. (**A**) Representative western blot for MnSOD, VDAC1 was used as internal control; (**B**) Relative protein level of MnSOD/VDAC1. Values not sharing a common letter (a, b, c, d, e, f) differ significantly at *p* < 0.05. SP, synthetic pentapeptide; MP, mixed corn peptides. Numbers 1–6 indicate groups of Control, Model, SP-10 mg/kg bw, SP-30 mg/kg bw, MP-50 mg/kg bw and MP-200 mg/kg bw, separately.

MnSOD located in the mitochondria is believed to be associated with oxidative stress. As shown in Figure 4, when compared with the control, the model group had a significant decrease (*p* < 0.05) in the relative protein level of MnSOD in the mitochondria, whereas every peptide-treated groups showed a significant increase over the model group in the level (*p* < 0.05), indicating the treatment effect of the synthetic pentapeptide and the mixed corn peptides.

#### 2.5.2. Effects of the Peptides on Intrinsic Pathway

**Figure 5:**
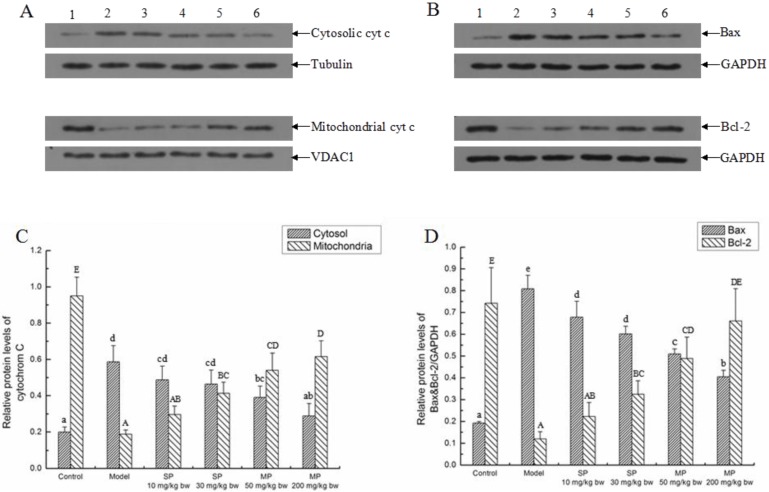
The relative protein expressions of cytochrome c, Bcl-2 and Bax in each group. Representative western blots for (**A**) cytochrome c; (**B**) Bcl-2 and Bax. Tubulin and VDAC1 were used as internal controls for cytosol and mitochondria, respectively. GAPDH was used as internal control for liver tissue; (**C**) Relative protein level of cytochrome c/Tubulin and cytochrome c/VDAC1; (**D**) Relative protein levels of Bax & Bcl-2/GAPDH. Values not sharing a common letter (a, b, c, d, e) in (**C**,**D**) differ significantly of cytochrome c/Tubulin and Bax/GAPDH at *p* < 0.05, separately. Values not sharing a common letter (A, B, C, D, E) in (**C**,**D**) differ significantly of cytochrome c/VDAC1 and Bcl-2/GAPDH at *p* < 0.05, separately. SP, synthetic pentapeptide; MP, mixed corn peptides. Numbers 1–6 indicate groups of Control, Model, SP-10 mg/kg bw, SP-30 mg/kg bw, MP-50 mg/kg bw and MP-200 mg/kg bw separately.

The relative protein levels of cyt c, Bcl-2 and Bax associated with intrinsic pathway were measured by Western blot and the results are shown in Figure 5. The relative protein level of cytosolic cyt c in model group was enhanced (increased by 1.94-fold) significantly compared with the control, while the relative protein level of that was significantly reduced in response to the mixed peptides (*p* < 0.05). Additionally, the relative protein level of the MP-200 mg/kg bw was reduced to the normal level. In contrast, the relative protein level of mitochondrial cyt c in model group was reduced significantly by ethanol compared with the control group (*p* < 0.05), and all the peptides-treated groups except the SP-10 mg/kg bw group were significantly higher than the model group. Similarly, the relative protein levels of Bax and Bcl-2 were increased (by 3.21-fold) and decreased (by 5.19-fold) significantly in the model group as compared to their control groups, respectively (*p* < 0.05). Compared with the model group, the relative protein levels of Bax in all the peptides-treated groups were significantly lower than that of the model group, and the relative protein levels of Bcl-2 in the groups of SP-30 mg/kg bw, MP-50 mg/kg bw and MP-200 mg/kg bw were significantly higher than that of the model group (*p* < 0.05). In addition, the relative protein levels of Bcl-2 in the groups of MP-200 mg/kg bw was reversed to the normal level. Generally, these effects were displayed in a dose-dependent manner.

#### 2.5.3. Effects of the Peptides on the Extrinsic Pathway

**Figure 6:**
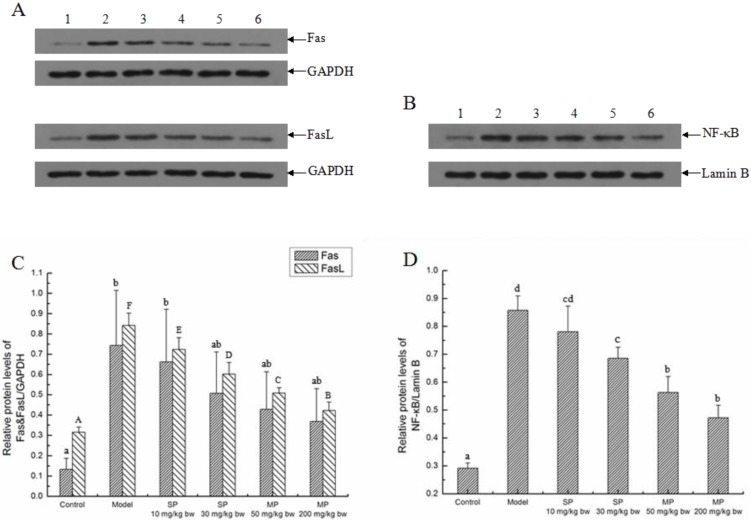
The relative protein expressions of Fas, FasL and NF-κB in each group. Representative western blots for (**A**) Fas and FasL; (**B**) NF-κB. GAPDH and LaminB were used as internal controls for liver tissue and nucleus, respectively; (**C**) Relative protein levels of Fas & FasL/GAPDH; (**D**) Relative protein level of NF-κB/LaminB. Values not sharing a common letter (a, b, c, d) in (**C**,**D**) differ significantly of Fas/GAPDH and NF-κB/LaminB at *p* < 0.05, separately. Values not sharing a common letter (A, B, C, D, E, F) in (**C**) differ significantly of FasL/GAPDH at *p* < 0.05. SP, synthetic pentapeptide; MP, mixed corn peptides. Numbers 1–6 indicate groups of Control, Model, SP-10 mg/kg bw, SP-30 mg/kg bw, MP-50 mg/kg bw and MP-200 mg/kg bw separately.

The relative protein levels of Fas, FasL and NF-κB associated with the death receptors-mediated extrinsic pathway are shown in Figure 6. The relative protein levels of Fas and FasL were elevated significantly in the model group as compared to their control groups (*p* < 0.05). Among these peptides-treated groups, the relative levels of FasL were significantly decreased in all the peptides-treated groups, while the relative levels of Fas significantly decreased and returned to the normal level only in the MP-200 mg/kg bw group. In addition, the relative levels of NF-κB significantly increased with the treatment of ethanol. However, they significantly decreased in response to the synthetic pentapeptide QLLPF and the mixed corn peptides compared with the model groups except for the pentapeptide of 10 mg/kg bw (*p* < 0.05).

#### 2.5.4. The Relative Expression Level of Caspase-3

**Figure 7:**
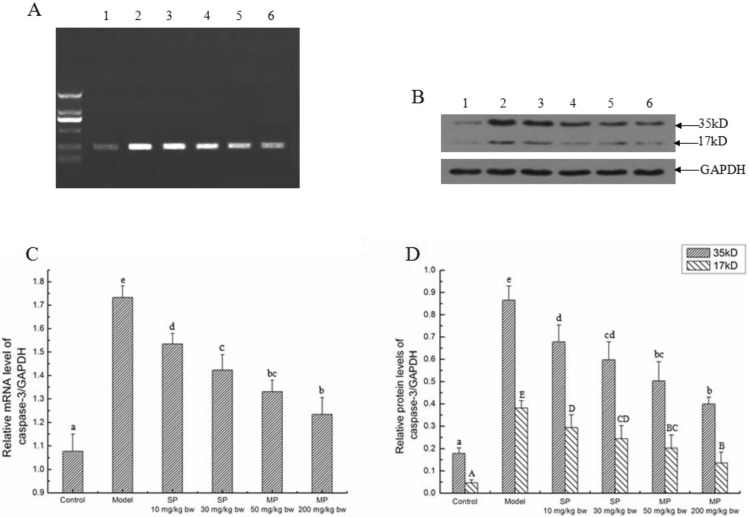
The relative mRNA and protein expressions of caspase-3 in each group. Representative (**A**) electrophoregram and (**B**) western blots for caspase-3. GAPDH was used as internal control; (**C**) Relative mRNA level of caspase-3/GAPDH; (**D**) Relative protein levels of caspase-3/GAPDH. Values not sharing a common letter (a, b, c, d, e) in (**C**,**D**) differ significantly of relative mRNA and protein levels of caspase-3/GAPDH at *p* < 0.05, separately. Values not sharing a common letter (A, B, C, D, E) in (**D**) differ significantly of relative protein level of cleaved caspase-3/GAPDH at *p* < 0.05. SP, synthetic pentapeptide; MP, mixed corn peptides. Numbers 1–6 indicate groups of Control, Model, SP-10 mg/kg bw, SP-30 mg/kg bw, MP-50 mg/kg bw and MP-200 mg/kg bw separately.

Caspase-3 is the dominant executioner of programmed cell death downstream. The mRNA and protein expression levels of caspase-3 are shown in Figure 7. The relative mRNA expression of caspase-3 and the relative protein expression of cleaved caspase-3 were significantly enhanced in response to ethanol in the model group (*p* < 0.05). In contrast, the levels of the synthetic pentapeptide QLLPF and the mixed corn peptides groups were significantly reduced compared with the model groups (*p* < 0.05).

#### 2.5.5. Effect of Peptides on Apoptosis

**Figure 8:**
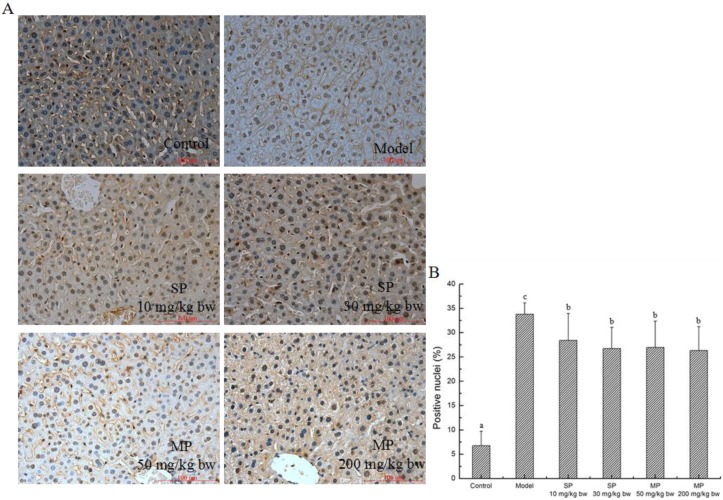
Detection of apoptotic hepatocytes by TUNEL assay in each group. (**A**) Representative images from each group and various administrations are shown at 400× magnifications; (**B**) TUNEL positive cells ratio. Values not sharing a common letter (a, b, c) differ significantly at *p* < 0.05. SP, synthetic pentapeptide; MP, mixed corn peptides.

In the present study, TUNEL staining was used to determine the effects of corn peptides on apoptosis, by locating apoptotic nuclei (Figure 8). As shown in Figure 8A, compared with the control group, a significant number of apoptotic cell nuclei were found in the model group. Interestingly, ballooning degeneration and apoptotic nuclei were both existing in the model group. In contrast, treatment with the pentapeptide and the mixed corn peptides led to significantly decreased percentages of positive nuclei compared with the model group (Figure 8B, *p* < 0.05). However, there was no significant difference in the number of apoptotic cells in the mixed corn peptides and pentapeptide-treated groups.

## 3. Discussion

It is known that acute and chronic alcoholism can cause liver injury. The present study investigated the effects of the pentapeptide QLLPF and the mixed corn peptides on ethanol-induced hepatocellular apoptosis in mice. The pentapeptide was identified from the mixed corn peptides mentioned and then synthesized. In our previous study, we analyzed the peptide sequences with high abundance and obtained information about several peptide sequences by HPLC-MS/MS coupled with the peptide sequence retrieval in the MS-MS online database (Table S1). According to amino acid analysis, the purity of the mixed corn peptides was calculated as 91.70%, with Glu, Leu, Ala and Pro accounting for a large proportion at 21.73%, 17.03%, 8.68% and 8.03%, respectively [18]. It is reported that branched-chain amino acids are generally associated with the amino-acid metabolism in contracting skeletal muscle and supplying pyruvic acid [28]. In the long-term CP ingestion and ethanol loading experiment, Yamaguchi *et al.* reported that the plasma alanine concentration was elevated by not only its supply from CP ingestion but also its release from the skeletal muscle as a result of metabolizing branched-chain amino acids, especially leucine [29]. Chen and Dickman demonstrate that the ability of Pro to scavenge intracellular ROS and inhibit ROS-mediated apoptosis may be an important and broad-based function of this amino acid in responding to cellular stress, in addition to its well established role as an osmolyte [30]. The three known metabolic enzyme systems that participate in the oxidation of alcohol all lead to overproduction of reactive oxygen species. Therefore, considering the amino acid composition of the peptides and the high frequency in the protein sequence, we chose QLLPF for the further study. However, it is worth mentioning that the other peptides still need to be investigated and some research has been conducted. In the present study, we found that both QLLPF and the mixed corn peptides can mitigate the liver injury through the evaluation of biochemical indices, improve the expressions of apoptotic-related proteins and ameliorate the hepatocyte apoptosis.

It is believed that the levels of AST and ALT in serum are elevated in acute ethanol-induced liver injury, along with the formation of lipid radicals and the depletion of GSH. As is evident from our results, ethanol consumption leads to liver injury characterized by these aforementioned biochemical parameters. However, when compared with the model group, these values were reversed with the treatment of the two peptides. The ability of alcohol to promote oxidative stress and the role of free radicals in alcohol-induced tissue injury clearly are important research areas, because such information may be of major therapeutic significance in attempts to prevent or ameliorate alcohol’s toxic effects, e.g., by antioxidants, iron chelators, inhibitors of CYP2E1 or of cytokine production/actions, and GSH replenishment [31]. According to a previous study [25], l-theanine significantly inhibited the increase of ALT, AST and MDA and the decrease of GSH stimulated by ethanol in mice, and it can be further speculated that l-theanine prevented ethanol-induced liver injury through enhancing hepatocyte antioxidant abilities. Similar results were also reported by Je *et al.* [32], who found the peptic hydrolysate from salmon pectoral fin protein byproducts had a hepatoprotective effect on ethanol-induced oxidative stress in Sprague-Dawley rats by examining the levels of ALT, AST, MDA, GSH, SOD and GPx. In our previous studies, Yu [18] also reported the antioxidant capability of the corn peptides *in vitro* and *in vivo*. Based on the values of MDA and GSH, and the expression of MnSOD, it can be concluded that the protective effect of the synthetic pentapeptide and the mixed corn peptides is related with the attenuation of the oxidative stress. The main metabolic pathway involved in the biotransformation of ethanol is oxidation into acetaldehyde, and this process uses NAD^+^ and is primarily achieved by alcohol dehydrogenase [33]. However, the studies on the activities of ADH and ALDH are not so consistent [34,35]. In our study, the expressions of ADH and ALDH in the model group were suppressed a lot more than the control group, which is in agreement with that reported by Sun *et al.* [34]. The decrease of ADH and ALDH levels indicated that the metabolism of ethanol and acetaldehyde was affected, whereas the peptides treatment could accelerate the clearance of toxic substances.

As a highly programmed, genetically controlled form of cell death, apoptosis is essential for the maintenance of development and homeostasis in multicellular organisms by eliminating superfluous or unwanted cells [36]. However, this balance will be disturbed in an injury induced by chemicals and ethanol is one of such chemicals. There are two cytokines that strongly influence the apoptotic process: TNF-α and TGF-β1 [37]. In the present study, we found that serum TNF-α and hepatic TGF-β1 were significantly elevated in the ethanol-induced injury, but the two cytokines decreased when treated with QLLPF and the mixed corn peptides.

We also detected the expressions of key proteins associated with apoptosis. Mitochondria are central to the life of eukaryotic cells and also play a key role in the pathways to cell death [38]. Production of reactive oxygen species may lead to mitochondrial damage that produces a leak of cyt c, which activates caspases and causes apoptosis [24]. Some research demonstrated that the mitochondria-mediated pathway of apoptosis is not only explained by a mere “loss of function” resulting in a bioenergetic defect, but also by a regulated effector mechanism involving cyt c release into the cytosol [39]. Additionally, Bcl-2 and Bax are also associated with the intrinsic pathway. Bcl-2 exerts anti-apoptotic effects, while Bax exerts pro-apoptotic effect [40]. In the present study, when compared with the model group, the synthetic pentapeptide and the mixed corn peptides can inhibit the leaking of cyt from mitochondria, down-regulate the expression of Bax and up-regulate the expression of Bcl-2. It was reported that rice protein hydrolysate attenuated the apoptosis of myocardiocytes H9c2 through the Bcl-2/Bax pathway during H_2_O_2_ challenge [41], which is another example to suggest that protein hydrolysate has the effect of inhibiting apoptosis. Some reports suggested that ethanol-induced oxidative stress mediates the MPT, and the MPT is essential for the induction of the release of mitochondrial cyt c and the caspase activation of ethanol [9]. Hence, we also measured the expression of MnSOD, the important antioxidant in mitochondria. As expected, the expression of MnSOD was down-regulated along with the release of mitochondrial cyt c in the model group, whereas it was up-regulated when treated with the two peptides. From these results, it can be concluded that the pentapeptide QLLPF and the mixed corn peptides may suppress apoptosis by regulating the intrinsic pathway.

In addition, a dual mechanism model has also been reported for hepatocyte apoptosis: one is MPT, and the other is the up-regulation of FasL [10]. Fas and FasL are the important members in death receptors-mediated extrinsic pathway [42]. The combination of Fas and FasL can induce apoptosis by starting the signal transduction and activating caspase proteases [43]. NF-κB is best known for the key role in normal immune and inflammatory responses, but it is implicated in the control of cell proliferation, differentiation, apoptosis and oncogenesis [44]. A study showed that inhibition of NF-kB sensitized HaCaT keratinocytes to TNF-induced apoptosis [45]. Feng *et al.* found that the NF-κB mediated the induction of Fas and FasL as well as cellular apoptosis induced by Microcystin-LR in HepG2 cells [46]. In this study, Fas, FasL and NF-κB were up-regulated when treated with ethanol, but the up-regulation was inhibited by the pentapeptide QLLPF and the mixed corn peptides, suggesting that the two peptides might exert the protective effect of ethanol-induced injury through participating in the two main signaling pathways of apoptosis.

As the dominant executioner of programmed cell death downstream, caspase-3 can be activated by both intrinsic pathways and extrinsic pathways. In the present study, caspase-3 was up-regulated by the stimulation of ethanol at both mRNA and protein expression levels. Compared with the model group, the levels were significantly down-regulated in response to QLLPF and the mixed corn peptides. From the perspective of protein expression, it is obvious that QLLPF did not show stronger effects than mixed corn peptides in the longer hepatoprotective experiment. Compared with QLLPF, the mixed corn peptides not only contain QLLPF, but also many other kinds of peptides. Although the content of QLLPF in mixed corn peptides group is lower than the QLLPF group, it still has a better effect of regulating proteins. Hence, we speculate that the compositions in the mixed peptides may exert a synergistic effect against hepatic injury under the experimental conditions, and QLLPF is an important contributor in the mixed peptides. The TUNEL assay results also indicated significant inhibition of hepatocyte apoptosis when treated with the peptides. However, as we can see from the number of apoptotic cells, it was not attenuated in a dose dependent manner. We hold the view that there are some other proteins related to apoptosis [5]; there probably exists other protein-mediated pathways. Maybe the comprehensive influence of every related protein led to the result. Nevertheless, based on statistical analysis, TUNEL-positive cells in peptides-treated groups were significantly reduced compared to the model group. Moreover, the key proteins in the suggested apoptosis pathways were reversed in response to the peptides, and some of the proteins have returned to normal levels. Therefore, our results suggest that the peptides can inhibit the hepatocyte apoptosis induced by ethanol.

## 4. Materials and Methods

### 4.1. Materials and Reagents

Alanine aminotransferase (ALT), aspartate aminotransferase (AST), malonaldehyde (MDA), glutathione (GSH), and kits were obtained from Nanjing JianCheng Bioengineering Institute (Nanjing, China). Elisa detection kits of tumor necrosis factor (TNF)-α and transforming growth factor (TGF)-β1 were obtained from Elabscience Biotechnology Co., Ltd. (Wuhan, China). The pentapeptide QLLPF was customarily synthesized by AoBo Corp (Shanghai, China) with purity >95%. All other chemicals, unless otherwise specified, were of analytical grade and purchased from Sinopharm Chemical Reagent Co., Ltd. (Shanghai, China).

### 4.2. Preparation of Concentrated Corn Protein and Corn Peptides

The concentrated corn protein was prepared according to the method of Guo *et al.* [17]. Briefly, protein extraction solvent was prepared by mixing 0.1 M NaOH and 95% EtOH at 45:55 ratio (*v*/*v*). The corn gluten meal was then soaked in this extraction solvent at 1:15 ratio (*w*/*v*) in 50 °C water bath for 2 h. The sample was then centrifuged (25 °C, 2280× *g*) for 10 min. After centrifugation, the separated supernatant was reconstituted with equal volume of distilled water, and the pH of this solution was further adjusted to 6.3, the pI of corn protein. A 2% NaCl solution was added to the above sample solution at the 1:5 ratio (*v*/*v*) for 1 h. After the final centrifugation (25 °C, 2280× *g*) for 10 min, the precipitate was dried under 40 °C air, and then sieved through an 80# mesh sieve. The process of producing corn peptides was carried out according to the method of Yu *et al.* [18]. Briefly, 4% concentrated corn protein suspensions (*w*/*v*) were heated at 90–100 °C for 30 min. The temperature of suspensions was then dropped to 55 °C, and the pH value was adjusted to 8.0 (the optimum pH of alcalase). The corn protein solution was then hydrolyzed by alcalase for 5 h, with an enzyme to substrate ratio of 0.8% (*w*/*w*). The pH of the reaction mixture was maintained at pH 8.0 by addition of 1.0 M sodium hydroxide. The reaction was terminated by a 10 min boiling treatment, and the final pH was adjusted to 7.0. The hydrolysate was transferred to an UF-membrane system (Prepscale TFF system 230 V, Millipore, Billerica, MA, USA), composed of 1.6 L/min pump and a tangential flow filter cartridge (PLCC, Millipore). The molecular weight cutoff (MwCO) of the regenerated cellulose membranes was 5 KDa with an effective membrane area of 0.09 m^2^. A fraction of the CPs (Mm < 5 kDa; namely mixed peptides) was concentrated and lyophilized for further tests.

### 4.3. Animal and Experimental Design

Kunming male mice (18–22 g) were obtained from Hubei Laboratory Animal Research Center (Wuhan, China). All mice were kept in stainless steel wire-bottomed cages with free access to food and water under standard environmental conditions of 22 °C and dark/light cycle. Animals were weighed every day, and measurements of daily food consumption were recorded.

The animals were cared for and handled in accordance with Regulation No. 5 of the Standing Committee of Hubei People’s Congress and the research was approved (HBAC20131018) by the ethics committee of Huazhong Agricultural University, Wuhan, China.

For the prevention experiment of peptides on alcohol-induced liver injury, mice (10/group) were randomly divided into the six groups: control group, model group, two synthetic pentapeptide groups (the dosages are 10 and 30 mg/kg bw, respectively) and two mixed corn peptides groups (the dosages are 50 and 200 mg/kg bw, respectively). Mice were pretreated with peptides dissolved in saline for one week through a gavage. During this time, those in the control and model groups received the equal volumes of saline. The doses were referred to the preliminary studies [18,27] and pre-test (including the mRNA and protein expressions of caspase-3). Peptides were administered intragastrically once daily for 3 weeks; food intake of mice were recorded every day. Excluding the mice in the control group, the other animals were initially administered intragastrically with 50% (*v*/*v*) ethanol 8 mL/kg/day 4 h after the doses of peptides/saline for 1 week following by an increasing intake of ethanol up to 10 mL/kg/day for the remaining 1 week. After the last administration, all groups were fasted for 16 h, then they were sacrificed for blood samples, and the serum was collected for biochemical assays. The livers were weighed, some were made into 10% of liver tissue homogenates for biochemical assays, some were fixed in 10% neutral buffered formalin solution for TUNEL assay, and some were stored at −80 °C for Western blot and real-time PCR assay.

### 4.4. Biochemical Parameters of Blood and Liver Analysis

The blood was centrifuged at 2500 rpm for 10 min at 4 °C. The serum ALT and AST activities as well as the contents of GSH and MDA in liver homogenate were determined by using the detection kits according to the manufacturer’s instructions.

### 4.5. Cytokines Analysis

The serum TNF-α and the liver TGF-β1 were determined by the Elisa detection kits according to the manufacturer’s instructions.

### 4.6. Western Blot Analysis

Liver tissue was lysed by RIPA Lysis Buffer (containing 50 mM Tris, pH 7.4/150 mM NaCl/1% Triton X-100/1% sodium deoxycholate/0.1% SDS/1 mM sodium orthovanadate/10 mM sodium fluoride/2 mM EDTA/10 μg/mL leupeptin/1 mM phenylmethylsulfonyl fluoride, Beyotime Biotechnology, Wuhan, China), equal amounts of proteins (50 μg) from the cytosolic fraction, mitochondrial fraction, and liver tissue were separated by SDS-polyacrylamide gel electrophoresis using 12% or 10% polyacrylamide gels and then transferred onto a polyvinylidene fluoride membranes (Millipore) for 1 h with blotting buffer. The membranes were blocked for 2 h with TBST (10 mM Tris-HCl, pH 8.0/150 mM NaCl/0.1% Tween20) containing 5% non-fat milk at room temperature. They were incubated with the primary antibody overnight at 4 °C. Antibodies against Bax (1:800) and Bcl-2 (1:800) were purchased from Cell Signaling Technology (Beverly, MA, USA); and FasL (1:200) was purchased from Abcam Biotechnology (Cambridge, MA, USA,); cyt c (1:1000), caspase-3 (1:600), Fas (1:500), NF-κB (1:1000) were purchased from Proteintech Group (Proteintech Group, Inc., Wuhan, China). After six 5-min washes in TBST, the membranes were incubated with horseradish peroxidase-conjugated secondary antibody (1:50000) (Zhongshan Jinqiao Biological Technology Co., Ltd., Beijing, China) at room temperature for 2 h. After six 5-min washes with TBST, the membranes were then incubated with ECL substrate solution (Thermo Fisher Scientific Inc., Waltham, MA, USA) for several minutes. The excess liquid substrate solution was removed after the brand was obvious and the X-ray film was developed and fixed in turn with developing fixing kit (Wuhan Biobuffer Biotech Service Co., Ltd., Wuhan, China).

The relative contents of protein in liver tissue, cytosol, mitochondria and nucleus were expressed as the ratio of protein to glyceraldehyde-3-phosphate dehydrogenase (GAPDH) (Hangzhou Xianzhi Biological Technology Co., Ltd., Hangzhou, China), Tubulin (Beijing Biosynthesis Biotechnology Co., Ltd., Beijing, China), voltage dependent anion channel (VDAC) (Santa Cruz Biotechnology, Inc., Dallas, TX, USA) and LaminB (Proteintech Group, Inc., Wuhan, China), respectively.

### 4.7. Real-Time (RT) PCR Analysis

The relative transcription levels of caspase-3 were measured using quantitative RT-PCR. The GAPDH was used as the internal control. The quantitative RT-PCR was done using an ABI ViiA7 Real-Time PCR System. The 2^−ΔΔ*C*t^ method was used for relative quantification analysis. All data for expression levels determined by quantitative RT-PCR were based on three biological samples with three technical replications.

### 4.8. Transferase dUTP Nick End Labeling (TUNEL) Assay

The liver tissues embedded in paraffin were sectioned for the TUNEL assay with a commercial TUNEL Apoptosis Assay Kit (Roche Applied Science, Indianapolis, IN, USA). Briefly, the tissue section was dewaxed by washing in xylene and rehydrated through a graded series of ethanol and double distilled water, and then was incubated for 20 min at 37 °C with Proteinase K working solution. After that, the slides were incubated with TUNEL reaction mixture for 60 min at 37 °C in a humidified atmosphere in the dark. Converter-POD was then added on the sample and the slide was incubated in a humidified chamber for another 30 min at 37 °C. Afterwards, the slides were incubated with diaminobenzidine (DAB) substrate solution for 5 min at room temperature in the dark. Sample was counterstained by hematoxylin, rinsed by water and differentiated by 1% hydrochloric acid alcohol solution for a few seconds. Finally, the slides were analyzed by light microscope. For each slide, 10 microscopic fields were randomly chosen for statistical analysis.

The apoptotic index was calculated using the following formula.

(1) Apoptotic index (100%) = (number of TUNEL positive cell nuclei/number of total cell nuclei) × 100%

### 4.9. Statistical Analysis

Analysis of variance was performed using the SPSS program (SPSS version 19). Differences among the mean values were established using Duncan Multiple Range Test at *p* < 0.05. Values are expressed as mean ± SD.

## 5. Conclusions

In conclusion, the mixed corn peptides and the corn-derived peptide QLLPF can inhibit the hepatocyte apoptosis induced by ethanol. The mechanisms of corn peptides to inhibit hepatocyte apoptosis are regulating Bcl-2 family proteins, inhibiting the release of cyt c into the cytosol, regulating the cell surface receptor Fas, its ligand FasL and the important nuclear factor NF-κB, and preventing the activation of caspase-3. Specifically, the corn peptides may suppress the hepatocyte apoptosis by participating in both the intrinsic and extrinsic pathways, which is likely to be an important mechanism for preventing alcoholic liver injury. The study provides some insights into understanding the features of corn peptides in exerting hepatoprotective effect and enriching their mechanisms, which may facilitate the application of corn peptides in the future.

## Supplementary Materials

Supplementary materials can be found at http://www.mdpi.com/1422-0067/16/09/22062/s1.

## Abbreviations

ACE-I: angiotensin I converting enzyme; ADH: alcohol dehydrogenase; ALDH: aldehyde dehydrogenase; ALT: alanine aminotransferase; AST: aspartate aminotransferase; Bax: Bcl-2-associated X protein; Bcl-2: B-cell lymphoma-2; CPs: corn peptides; cyt c: cytochrome c; DAB: diaminobenzidine; Fas: factor associated suicide; FasL: Fas ligand; GAPDH: glyceraldehyde-3-phosphate dehydrogenase; GSH: glutathione; MDA: malonaldehyde; MPT: membrane permeability transition; QLLPF: Gln-Leu-Leu-Pro-Phe; TNF: tumor necrosis factor; TGF: transforming growth factor; caspase: cysteinyl aspartate specific proteinase; VDAC: voltage dependent anion channel; NF-κB: nuclear factor-κB; TUNEL: terminal deoxynucleotidyl transferase dUTP nick end labeling.
