# Supplementary material for: Inhibition of Hepatocyte Apoptosis: An Important Mechanism of Corn Peptides Attenuating Liver Injury Induced by Ethanol
Source: Int J Mol Sci. 2015 Sep 11;16(9):22062–80. doi: 10.3390/ijms160922062 (PMC4613297; doi:10.3390/ijms160922062)
Supplement: Supplementary file 1 [file ijms-16-22062-s001.pdf]

Supplementary Information

Table S1. Data summary of mixed corn peptide analysis.

**Retention Time (min)**	**Molecular Ion [M + H]**	**Sequence of Peptides**
17.8	605.3	KPFIT/KPFLT
29.4	617.3	QLLPF
33.7	600.3	HSIPF/SHLPF
35.6	634.3	EEIPF
